# Supplementary material for: LMSM: A modular approach for identifying lncRNA related miRNA sponge modules in breast cancer
Source: PLoS Comput Biol. 2020 Apr 23;16(4):e1007851. doi: 10.1371/journal.pcbi.1007851 (PMC7200020; doi:10.1371/journal.pcbi.1007851)
Supplement: S1 File — Supplementary file. (DOCX) [file pcbi.1007851.s009.docx]

**LMSM: a modular approach to identify lncRNA related miRNA sponge modules in breast cancer**

Junpeng Zhang^1,2,*,†^, Taosheng Xu^3,†^, Lin Liu^4^, Wu Zhang^5^, Chunwen Zhao^2^, Sijing Li^2^, Jiuyong Li^4^, Nini Rao^1,*^ and Thuc Duy Le^4,*^

^1^School of Life Science and Technology, University of Electronic Science and Technology of China, Chengdu, Sichuan 610054, China

^2^School of Engineering, Dali University, Dali, Yunnan 671003, China

^3^Institute of Intelligent Machines, Hefei Institutes of Physical Science, Chinese Academy of Sciences, Hefei, Anhui 230031, China

^4^School of Information Technology and Mathematical Sciences, University of South Australia, Mawson Lakes, SA 5095, Australia

^5^School of Agriculture and Biological Sciences, Dali University, Dali, Yunnan 671003, China

*To whom correspondence should be addressed. Email: zhangjunpeng_411@yahoo.com

Correspondence may also be addressed to Nini Rao. Email: raonn@uestc.edu.cn

Correspondence may also be addressed to Thuc Duy Le. Email: thuc.le@unisa.edu.au

^†^The authors wish it to be known that, in their opinion, the first two authors should be regarded as Joint First Authors.

In the supplementary file, we will show the robustness of the LMSM workflow. In the LMSM workflow, the SGFA method [1] instead of the WGCNA method [2] is used to identify lncRNA-mRNA co-expression modules. The SGFA method is extended from the group factor analysis (GFA) method [3-5], and it can reliably infer ncRNA-mRNA co-expression modules from multiple data sources.

**Table A. BRCA-related LMSM modules**. *L*_2_ is the number of BRCA genes in each LMSM module, *K*_2_ represents the number of genes in each LMSM module, the number of BRCA genes in the dataset (*M*_2_) is 4819, and the number of genes in the dataset (*N*_2_) is 31055. The method of identifying lncRNA-mRNA co-expression modules is the SGFA method.

| **Module ID** | ***L*_2_** | ***K*_2_** | ***p*-value** |
| --- | --- | --- | --- |
| LMSM 3 | 35 | 129 | 5.02E-04 |
| LMSM 24 | 58 | 225 | 4.67E-05 |
| LMSM 37 | 54 | 256 | 1.05E-02 |

**Table B**. **Survival analysis of LMSM modules in BRCA**. HRlow95 and HRup95 represent the lower and upper of 95% confidence interval of HR, respectively. The identified LMSM modules can distinguish the high and the low risk BRCA samples. The method of identifying lncRNA-mRNA co-expression modules is the SGFA method.

| **Module ID** | **Chi-square** | ***p-*value** | **HR** | **HRlow95** | **HRup95** |
| --- | --- | --- | --- | --- | --- |
| LMSM 1 | 44.73 | 0 | 4.63 | 2.85 | 7.53 |
| LMSM 2 | 137.90 | 0 | 12.82 | 7.54 | 21.82 |
| LMSM 3 | 79.11 | 0 | 13.35 | 8.32 | 21.44 |
| LMSM 4 | 130.22 | 0 | 15.18 | 9.08 | 25.40 |
| LMSM 6 | 116.65 | 0 | 13.66 | 8.26 | 22.60 |
| LMSM 7 | 123.68 | 0 | 9.54 | 5.58 | 16.33 |
| LMSM 8 | 77.43 | 0 | 12.93 | 8.06 | 20.73 |
| LMSM 9 | 118.29 | 0 | 8.82 | 5.15 | 15.08 |
| LMSM 10 | 114.46 | 0 | 10.67 | 6.34 | 17.95 |
| LMSM 11 | 78.48 | 0 | 7.94 | 4.86 | 12.98 |
| LMSM 12 | 113.78 | 0 | 9.60 | 5.72 | 16.12 |
| LMSM 13 | 126.32 | 0 | 13.68 | 8.17 | 22.90 |
| LMSM 14 | 137.76 | 0 | 13.81 | 8.14 | 23.43 |
| LMSM 15 | 79.19 | 0 | 11.91 | 7.41 | 19.16 |
| LMSM 16 | 131.02 | 0 | 15.20 | 9.09 | 25.43 |
| LMSM 17 | 84.39 | 0 | 9.04 | 5.55 | 14.70 |
| LMSM 18 | 125.92 | 0 | 11.18 | 6.61 | 18.91 |
| LMSM 19 | 117.06 | 0 | 10.95 | 6.49 | 18.49 |
| LMSM 20 | 102.56 | 0 | 10.61 | 6.43 | 17.49 |
| LMSM 21 | 126.39 | 0 | 13.59 | 8.12 | 22.73 |
| LMSM 22 | 126.92 | 0 | 14.70 | 8.82 | 24.50 |
| LMSM 23 | 137.89 | 0 | 13.66 | 8.06 | 23.14 |
| LMSM 24 | 114.30 | 0 | 9.53 | 5.63 | 16.14 |
| LMSM 26 | 112.30 | 0 | 10.63 | 6.39 | 17.70 |
| LMSM 27 | 147.92 | 0 | 13.60 | 7.93 | 23.33 |
| LMSM 28 | 134.24 | 0 | 10.93 | 6.34 | 18.86 |
| LMSM 29 | 103.88 | 0 | 6.12 | 3.39 | 11.06 |
| LMSM 30 | 122.37 | 0 | 14.25 | 8.58 | 23.67 |
| LMSM 31 | 108.68 | 0 | 10.69 | 6.42 | 17.82 |
| LMSM 32 | 103.80 | 0 | 10.66 | 6.46 | 17.59 |
| LMSM 33 | 96.27 | 0 | 8.42 | 5.04 | 14.06 |
| LMSM 34 | 133.44 | 0 | 12.56 | 7.40 | 21.31 |
| LMSM 35 | 116.80 | 0 | 12.07 | 7.23 | 20.15 |
| LMSM 36 | 133.40 | 0 | 13.36 | 7.91 | 22.57 |
| LMSM 37 | 132.54 | 0 | 11.31 | 6.60 | 19.37 |
| LMSM 38 | 144.22 | 0 | 12.00 | 6.94 | 20.72 |
| LMSM 39 | 146.36 | 0 | 10.85 | 6.15 | 19.15 |
| LMSM 40 | 100.87 | 0 | 10.00 | 6.05 | 16.55 |
| LMSM 41 | 110.26 | 0 | 11.45 | 6.89 | 19.01 |
| LMSM 42 | 119.99 | 0 | 11.94 | 7.16 | 19.91 |
| LMSM 43 | 123.84 | 0 | 11.50 | 6.85 | 19.31 |
| LMSM 44 | 138.95 | 0 | 11.81 | 6.85 | 20.35 |
| LMSM 45 | 111.48 | 0 | 10.59 | 6.36 | 17.62 |
| LMSM 46 | 188.23 | 0 | 13.08 | 7.17 | 23.84 |
| LMSM 47 | 122.63 | 0 | 11.84 | 7.03 | 19.94 |
| LMSM 48 | 141.91 | 0 | 14.17 | 8.33 | 24.12 |
| LMSM 49 | 150.27 | 0 | 14.76 | 8.62 | 25.26 |
| LMSM 50 | 119.12 | 0 | 13.13 | 7.88 | 21.89 |
| LMSM 51 | 105.11 | 0 | 10.92 | 6.61 | 18.05 |

**Table C.** **Experimentally validated lncRNA-related miRNA sponge interactions**. The method of identifying lncRNA-mRNA co-expression modules is the SGFA method.

| **Module ID** | **Validated lncRNA- related miRNA sponge interactions** |
| --- | --- |
| LMSM 2 | *LINC00052*:*NTRK3* |
| LMSM 16 | *PVT1*:*VEGFC* |
| LMSM 27 | *SNHG7*:*CCND1* |
| LMSM 41 | *H19*:*AKT2* |

**Table D. Comparison results between LMSM and GC**.

| **Method** | **%BRCA-related modules** | **%Module biomarkers** | **Mean *Subset accuracy*** | **Mean *Hamming loss*** | **#Validated interactions** |
| --- | --- | --- | --- | --- | --- |
| LMSM | 5.88% | **96.08%** | **0.6921** | **0.1135** | **4** |
| GC | **32.41%** | 66.67% | 0.6586 | 0.1319 | 2 |

**B**

**A**

**Fig A. Heatmap of the enrichment scores of BRCA subtype-specific LMSM modules in five BRCA subtype samples**. (A) Up-regulated BRCA subtype-specific LMSM modules. (B) Down-regulated BRCA subtype-specific LMSM modules. The method of identifying lncRNA-mRNA co-expression modules is the SGFA method.

**A**

**B**

**Fig B. Overlaps and differences between predicted miRNA-target interactions by LMSM and other methods**. (A) Predicted miRNA-mRNA interactions between LMSM and TargetScan, DIANA_microT_CDS, starBase, miRWalk. (B) Predicted miRNA-lncRNA interactions between LMSM and starBase, DIANA_LncBase. Each column corresponds to an exclusive intersection that includes the elements of the sets denoted by the dark or red circles, but not of the others. The overlap size between different methods denotes exclusive overlaps, i.e. the overlap set not in a subset of any other overlap set. The method of identifying lncRNA-mRNA co-expression modules is the SGFA method.

References

1. Bunte K, Leppäaho E, Saarinen I, Kaski S. Sparse group factor analysis for biclustering of multiple data sources. Bioinformatics 2016; 32(16):2457-63. doi: 10.1093/bioinformatics/btw207.
2. Langfelder P, Horvath S. WGCNA: an R package for weighted correlation network analysis. BMC Bioinformatics 2008; 9:559. doi: 10.1186/1471-2105-9-559.
3. Klami A, Virtanen S, Leppäaho E, Kaski S. Group factor analysis. IEEE Trans Neural Netw Learn Syst 2015; 26(9):2136-2147. doi: 10.1109/TNNLS.2014.2376974.
4. Suvitaival T, Parkkinen JA, Virtanen S, Kaski S. Cross-organism toxicogenomics with group factor analysis. Syst Biomed 2014; 2(4):71-80. doi: 10.4161/sysb.29291.
5. Virtanen S, Klami A, Khan S, Kaski S. Bayesian group factor analysis. In: Lawrence,N. and Girolami,M. (eds), Proc. of the 15th International Conference on Artificial Intelligence and Statistics, 2012; pp. 1269-1277.
