# Supplementary material for: LMSM: A modular approach for identifying lncRNA related miRNA sponge modules in breast cancer
Source: PLoS Comput Biol. 2020 Apr 23;16(4):e1007851. doi: 10.1371/journal.pcbi.1007851 (PMC7200020; doi:10.1371/journal.pcbi.1007851)
Supplement: Attachment — Submitted filename: Response to Reviewers.docx [file pcbi.1007851.s010.docx]

Dear Editor and Reviewers,

Thank you very much for your comments on our manuscript, entitled “LMSM: a modular approach for identifying lncRNA related miRNA sponge modules in breast cancer” (Manuscript ID: PCOMPBIOL-D-19-01970). All the comments and suggestions are very helpful for improving the quality of the paper.

We have revised the manuscript by following the comments carefully. Our responses to all the comments, including the details on how the manuscript has been revised, are provided in the following Responses to Reviewers' Comments section.

**Responses to Reviewers' Comments**

**Reviewer #1:**

**Comment R1.1:** The paper describes a framework, LMSM, to identify LncRNA related MiRNA Sponge Modules from heterogeneous data. To understand the miRNA sponging activities in biological conditions, LMSM uses gene expression data to evaluate the influence of the shared miRNAs on the clustered sponge lncRNAs and mRNAs. This may be an important paper in the field.

**Response:** Thanks for the positive comment.

**Comment R1.2:** I do not see any control experiments. There is some overlap with previous results however a statistical significance of this overlap is not clear at all. There are many lncRNAs, some overlap is expected due to random reasons.

**Response:** Thanks for the comment. The random bias of our LMSM modules is probably caused by several factors including canonical correlation, sample number and the group of shared miRNAs. This bias can be adjusted by using a suitable null model for inferring statistical significance as suggested in (List M, Dehghani Amirabad A, Kostka D, Schulz MH. Large-scale inference of competing endogenous RNA networks with sparse partial correlation. Bioinformatics. 2019; 35(14): i596–i604. doi:10.1093/bioinformatics/btz314). Therefore, in this version, we add a null model to evaluate statistical significance of LMSM modules. In our null model, the null hypothesis is that the group of the shared miRNAs does not influence the canonical correlation between the group of lncRNAs and the group of mRNAs. The result of the hypothesis test shows that all of the identified LMSM modules are statistically significant.

In addition, based on the statistically significant LMSM modules, we have further found that the majority of LMSM modules are significantly implicated in BRCA by using two statistical significance analysis, including BRCA enrichment analysis and module survival analysis.

For your convenience, we extract the related paragraphs and present them in the following.

(In Section Evaluating statistical significance of LMSM modules)

To evaluate the statistical significance of LMSM modules, we adapt the null model method proposed in [25]. The null model method hypothesizes that the shared miRNAs do not affect the correlation between two genes, i.e. the sensitivity correlation (the difference between correlation and partial correlation) between two genes is 0, and has been successfully applied to evaluate statistical significance of ceRNA interactions. Similar to [25], LMSM is also adapted from the Sensitivity Correlation (SC) method [24]. Therefore, the null model method can be applied to evaluate the statistical significance of LMSM modules. In our null model, the null hypothesis is that the group of the shared miRNAs does not influence the canonical correlation between the group of lncRNAs and the group of mRNAs, i.e. *SCC_lncR-mR_* = 0. For each LMSM module, a group of lncRNAs or a group of mRNAs corresponds to a gene, and a group of the shared miRNAs corresponds to a miRNA in the null model. For obtaining more precise *p*-values, the number of datasets sampled is set to 1E+06 for the null model. Since the sampling procedure is computationally intensive, we use the pre-computed sets of covariance matrices in *SPONGE* R package [25] to build our null model. Based on the constructed null model, we can infer adjusted *p*-values (adjusted by Benjamini and Hochberg method [26]) for each LMSM module. A LMSM module with adjusted *p*-value less than 0.05 is regarded as a statistically significant module.

(In Section LMSM modules are all statistically significant)

In this section, by computing null-model-based *p*-values, we evaluate whether the identified LMSM modules are statistically significant or not. As a result, the adjusted *p*-values for the identified 17 LMSM modules (details can be seen in S3 Data) are all statistically significant (adjusted *p*-value = 1.00E-06). This result demonstrates that LMSM modules are all statistically significant.

**Comment R1.3:** There is no attempt to check biological relevance of the obtained module classification. I cannot understand a quality of the classification. May be some experiments will improve the paper.

**Response:** Thanks for the comment. In this version, to check the biological relevance of the discovered LMSM modules, we add a section to conduct multi-label classification analysis of LMSM modules in classifying BRCA subtypes. As a result, we have found that the classification performance of LMSM modules is significantly higher than baseline’s performance. The better performance than the baseline method indicates that LMSM modules are biological meaningful in classifying BRCA subtypes. For your convenience, we extract the related paragraphs and present them in the following.

(In Section Performance of LMSM modules in classifying BRCA subtypes)

In this section, to check the biological relevance of the discovered LMSM modules, we conduct module classification of BRCA subtypes. Here, classifying BRCA subtypes (LumA, LumB, Her2, Basal and Normal) is a multi-class classification (also known as a special case of multi-label classification). To understand the classification performance of the feature genes in each LMSM module, we apply a state-of-the-art multi-label learning strategy called Binary Relevance (BR) [33] implemented in the *utiml* R package [34] to conduct multi-label classification analysis. For the BR strategy, we use the Support Vector Machine (SVM) classifier [35] with default parameters implemented in *e1071* R package [36] as the base algorithm to build the multi-label model. We select two commonly used multi-label classification measures: *Subset accuracy* and *Hamming loss*, and conduct 10-fold cross-validation to evaluate the performance of each LMSM module. In this work, *Subset accuracy* denotes the percentage of correct predictions and *Hamming loss* is the fraction of wrong predictions to the total number of predictions. Higher values of *Subset accuracy* and smaller values of *Hamming loss* indicate better classification performance. In addition, for the evaluation, we use the baseline method in [37], a commonly used multi-label classification method as the baseline for comparison. The base algorithm of the baseline method is also the SVM classifier with default parameters implemented in *e1071* R package [36].

(In Section The performance of LMSM modules is significantly higher than baseline’s performance in classifying BRCA subtypes)

For the identified 17 LMSM modules, the average *Subset accuracy* and *Hamming loss* in classifying BRCA subtypes is 0.7547 and 0.0892, respectively (details can be seen in S6 Data), The *Subset accuracy* and *Hamming loss* of the baseline are 0.3800 and 0.2480, respectively. By using Welch’s *t*-test method, the *Subset accuracy* achieved using the 17 LMSM modules is significantly larger (better) than the *Subset accuracy* of the baseline (*p*-value < 2.20E-16), and the *Hamming loss* of the 17 LMSM modules is significantly smaller (better) than the *Hamming loss* of the baseline (*p*-value < 2.20E-16). The better performance than the baseline method indicates that LMSM modules are biological meaningful in classifying BRCA subtypes.

**Reviewer #2:**

**Comment R2.1:** In their manuscript "LMSM: a modular approach for identifying lncRNA related miRNA sponge modules in breast cancer", Zhang et al. present a novel method for investigating the competing endogeneous RNA effect of long non-coding RNAs (lncRNA) on the level of modules. Here LMSM differs from existing methods that focus on pair-wise considerations of lncRNA and mRNAs. The authors refer to this as miRNA sponge modular competition hypothesis. To obtain modules, LMSM uses the well established method WCGNA. Modules containing lncRNA as well as mRNAs (considered as two groups) are then evaluated using three criteria (i) significant number of shared miRNAs assessed using the hypergeometric test, (ii) canonical correlation analysis to assess the influence of the lncRNA group over the mRNA group, (iii) miRNA expression data is included to compute the partial-correlation based sensitivity correlation which was first introduced by Paci et. al. Sensitivity correlation is modified here for the use of partial canonical correlation to consider the effect of the lncRNA group to the mRNA group.

**Response:** Thanks for your constructive comments. In this version, we have further improved the work according to your detailed comments.

**Comment R2.2:** While it is appreciated that the authors released their source code on github, the software should also be documented, i.e. a README with installation instructions, dependencies and usage examples is needed.

**Response:** Thanks for the advice. In this version, to help users, we have updated the README file of LMSM with installation instructions, dependencies and usage examples.

**Comment R2.3:** The authors differentiate between competing RNAs such as lncRNA or circRNAs and the mRNAs as a target group. I do not agree with this view as it neglects the fact that mRNAs act as competitors as well. For example, a highly expressed protein-coding mRNA may sponge miRNAs to increase the availability of other mRNAs coding for important interacting proteins, thus allowing the formation of protein complexes. This limitation should be discussed more critically in the manuscript.

**Response:** Thanks for your advice. Indeed, as you said, mRNA can also sponge miRNAs to increase the availability of other mRNAs coding for important interacting proteins. In this version, we have clarified that other coding RNAs also include mRNAs in the “*A hypothesis on miRNA sponge modular competition*” section.

**Comment R2.4:** In the Methods, the authors state that "the available resources of lncRNAs are more abundant than those of other coding RNAs, circRNAs and pseudogenes". I do not understand this statement as clearly coding mRNAs are more abundant than lncRNAs.

**Response:** Sorry for the misleading description. What we meant to say was that the available resources of lncRNAs are more abundant than those of other non-coding RNAs (e.g. circRNAs and pseudogenes). In this version, we have clarified it.

**Comment R2.5:** In WCGNA, R2 is empirically set as 0.8. Can you elaborate what you mean by empirically?

**Response:** Thanks for the comment. The *R*^2^ (ranges from 0 to 1) is used to quantify the goodness of scale-free topology, and larger *R*^2^ values mean better scale-free topology. Normally, the *R*^2^ value larger than 0.8 in power law curve fit is ranked as good-level in the WGCNA method. Therefore, the desired minimum scale free topology fitting index *R*^2^ is set as 0.8 in this work. In this version, we have further clarified it in the section “*The LMSM framework*”.

**Comment R2.6:** In the third module assessment step, sensitivity correlation was used to quantify the sponging effect based on matched gene and miRNA expression data. We could recently show a major issue with sensitivity correlation is that it is biased by several factors including gene-gene correlation, sample number and number of shared miRNAs (List et al., https://doi.org/10.1093/bioinformatics/btz314). This bias can be adjusted for by using a suitable null model for inferring significance. Could this null model approach described in the above paper be adapted towards the use partial canonical correlation?

**Response:** Thanks for your advice. Yes, this bias can be adjusted by a null model described by the paper (List M, Dehghani Amirabad A, Kostka D, Schulz MH. Large-scale inference of competing endogenous RNA networks with sparse partial correlation. Bioinformatics 2019; 35(14): i596-i604. doi: 10.1093/bioinformatics/btz314.). In this version, we add a null model to evaluate the statistical significance of LMSM modules. In our null model, the null hypothesis is that the group of the shared miRNAs does not influence the canonical correlation between the group of lncRNAs and the group of mRNAs. For your convenience, we extract the related paragraphs and present them in the following.

(In Section Evaluating statistical significance of LMSM modules)

To evaluate the statistical significance of LMSM modules, we adapt the null model method proposed in [25]. The null model method hypothesizes that the shared miRNAs do not affect the correlation between two genes, i.e. the sensitivity correlation (the difference between correlation and partial correlation) between two genes is 0, and has been successfully applied to evaluate statistical significance of ceRNA interactions. Similar to [25], LMSM is also adapted from the Sensitivity Correlation (SC) method [24]. Therefore, the null model method can be applied to evaluate the statistical significance of LMSM modules. In our null model, the null hypothesis is that the group of the shared miRNAs does not influence the canonical correlation between the group of lncRNAs and the group of mRNAs, i.e. *SCC_lncR-mR_* = 0. For each LMSM module, a group of lncRNAs or a group of mRNAs corresponds to a gene, and a group of the shared miRNAs corresponds to a miRNA in the null model. For obtaining more precise *p*-values, the number of datasets sampled is set to 1E+06 for the null model. Since the sampling procedure is computationally intensive, we use the pre-computed sets of covariance matrices in *SPONGE* R package [25] to build our null model. Based on the constructed null model, we can infer adjusted *p*-values (adjusted by Benjamini and Hochberg method [26]) for each LMSM module. A LMSM module with adjusted *p*-value less than 0.05 is regarded as a statistically significant module.

(In Section LMSM modules are all statistically significant)

In this section, by computing null-model-based *p*-values, we evaluate whether the identified LMSM modules are statistically significant or not. As a result, the adjusted *p*-values for the identified 17 LMSM modules (details can be seen in S3 Data) are all statistically significant (adjusted *p*-value = 1.00E-06). This result demonstrates that LMSM modules are all statistically significant.

**Comment R2.7:** What is the source for the interactions of H19 and NEAT1? H19 and IGF2 for example share a complex relationship in imprinting that is not likely explainable by miRNA regulation. Thus, the results of module 2 should be seen critically.

**Response:** Thanks for the comment. The source of the experimentally validated miRNA sponge interactions used in this paper includes three databases of experimentally validated interactions, including miRSponge, LncCeRBase and LncACTdb v2.0. For the experimentally validated miRNA sponge interactions, the three databases provide existing literatures to support.

In Table 3, all of experimentally validated miRNA sponge interactions are supported by existing literatures. For example, after checking these databases, we found that the evidence for the interaction H19: IGF2 is from the reference (Kallen AN, Zhou XB, Xu J, et al. The imprinted H19 lncRNA antagonizes let-7 microRNAs. Mol Cell. 2013; 52(1):101–112. doi:10.1016/j.molcel.2013.08.027). The reference shows that H19 competes with IGF2 for sponging let-7b. Moreover, the evidence for the interaction NEAT1: EMP2 is from the reference (Wang Y, Wang C, Chen C, et al. Long non-coding RNA NEAT1 regulates epithelial membrane protein 2 expression to repress nasopharyngeal carcinoma migration and irradiation-resistance through miR-101-3p as a competing endogenous RNA mechanism. Oncotarget. 2017; 8(41):70156–70171.). The reference shows that NEAT1 competes with EMP2 for sponging miR-101-3p.

In addition, we have further carefully checked the results of Table 3. According to the three databases (miRSponge, LncCeRBase and LncACTdb v2.0), all of the validated lncRNA-related miRNA sponge interactions have supported references.

**Comment R2.8:** The main innovation of this method is that it infers miRNA sponge modules directly rather than first generating a comprehensive network which is then split into modules via graph clustering approaches. While this is indeed a promising strategy, the manuscript lacks a quantitative comparison to show advantages over the graph clustering strategy.

**Response:** Thanks for your advice. In this version, we have added a section “Comparison with graph clustering-based strategy” to compare LMSM with a graph clustering-based strategy. As there is no graph clustering-based strategy specifically designed for finding lncRNA related miRNA sponge modules, so we create a baseline Graph Clustering-based method (called GC in this paper) which uses well-known network construction and graph clustering methods. The GS method includes two steps: i) identifying lncRNA related miRNA sponge interaction network, and ii) identifying lncRNA related miRNA sponge modules from the identified network. In step 1, we adapt the well-cited Sensitivity Correlation (SC) method implemented in the *miRspongeR* R package to infer lncRNA related miRNA sponge interaction network. In step 2, we use the well-cited Markov cluster (MCL) algorithm to infer lncRNA related miRNA sponge modules.

The results show that, in terms of the percentage of BRCA-related modules, the percentage of module biomarkers in BRCA, the performance (mean *Subset accuracy* and mean *Hamming loss*) in classifying BRCA subtypes, and the number of validated lncRNA-related miRNA sponge interactions, LMSM performs better than the graph clustering strategy. For your convenience, we extract the related paragraphs and present them in the following.

(In Section Comparison with graph clustering-based strategy)

Graph clustering-based strategy [12-17] is an alternative approach to identifying lncRNA related miRNA sponge modules. As there is no graph clustering-based strategy specifically designed for finding lncRNA related miRNA sponge modules, so we create a baseline Graph Clustering-based method (called GC in this paper) which uses well-known network construction and graph clustering methods as described in the following. The GS method includes two steps: i) identifying lncRNA related miRNA sponge interaction network, and ii) identifying lncRNA related miRNA sponge modules from the identified network. In step 1, we adapt the well-cited Sensitivity Correlation (SC) method [24] implemented in the *miRspongeR* R package [56] to infer lncRNA related miRNA sponge interaction network. A lncRNA-mRNA pair is considered as an interacting pair in the network if they have significant sharing of the miRNAs, significant correlation and adequate sensitivity correlation. We require that the pairs must share at least 3 miRNAs and their sensitivity correlation (the difference between correlation and partial correlation) must be larger than 0.1. The statistically significance of the miRNA sharing and positive correlations are tested using hypergeometric test and Welch's *t*-test respectively, with a significant level at 0.05. In step 2, we use the well-cited Markov cluster (MCL) algorithm [57] to infer lncRNA related miRNA sponge modules. Here, each obtained cluster corresponds to a module. Each module should contain at least 2 sponge lncRNAs and 2 target mRNAs. In total, by using the GC method, we have obtained 108 lncRNA related miRNA sponge modules.

We compare LMSM and GC in terms of the percentage of BRCA-related modules, the percentage of module biomarkers in BRCA, the classification performance (mean *Subset accuracy* and mean *Hamming loss*) in classifying BRCA subtypes, and the number of validated lncRNA-related miRNA sponge interactions. As shown in Table 4, the comparison result indicates that LMSM always performs better than the GC method. The detailed results of the GC method can be seen in S8 Data.

**Comment R2.9:** Table 1, column N2 is superfluous; N2 can be mentioned once in the caption.

**Response:** Thanks for your advice. Corrected.

**Comment R2.10:** The interaction H19:TGFB1 is listed twice in Table 3.

**Response:** We have checked the Table 3 again. The interactions (*H19*:*TGFB1* and *H19*:*TGFBI*) are actually two different interactions. Perhaps the italic number “*1*” is very similar with the italic letter “*I*”.

We thank the editor and all reviewers for your comments, which have greatly helped us improve the quality of the paper.

Yours sincerely,

Junpeng Zhang & co-authors
